# The HMGB1/RAGE Pro-Inflammatory Axis in the Human Placenta: Modulating Effect of Low Molecular Weight Heparin

**DOI:** 10.3390/molecules22111997

**Published:** 2017-11-17

**Authors:** Cristian Zenerino, Anna Maria Nuzzo, Domenica Giuffrida, Marilisa Biolcati, Alessandra Zicari, Tullia Todros, Alessandro Rolfo

**Affiliations:** 1 Department of Surgical Sciences, University of Turin, 10126 Turin, Italy; cristian.zenerino@gmail.com (C.Z.); a.nuzzo@unito.it (A.M.N.); dgiuffrida@inwind.it (D.G.); marilisa.biolcati@unito.it (M.B.); tullia.todros@unito.it (T.T.); 2 Department of Experimental Medicine, Sapienza University of Rome, 00185 Rome, Italy; alessandra.zicari@uniroma1.it

**Keywords:** heparin, HMGB1, placenta, pre-eclampsia, receptor for advanced glycation end products (RAGE)

## Abstract

We evaluated whether physiological and pre-eclamptic (PE) placentae, characterized by exacerbated inflammation, presented alterations in pro-inflammatory High Mobility Group Box 1 (HMGB1) and its Receptor of Advanced Glycation End products (RAGE) expression. Moreover, we investigated, in physiological placental tissue, the ability of Low Molecular Weight Heparin (LMWH) to modify HMGB1 structural conformation thus inhibiting RAGE binding and HMGB1/RAGE axis inflammatory activity. HMGB1, RAGE, IL-6 and TNFα (HMGB1/RAGE targets) mRNA expression were assessed by Real Time PCR. HMGB1, RAGE protein levels were assessed by western blot assay. Physiological term placental explants were treated by 0.5 U LMWH for 24 or 48 h. HMGB1 and RAGE expression and association were evaluated in LMWH explants by RAGE immunoprecipitation followed by HMGB1 immunoblot. HMGB1 spatial localization was evaluated by immuofluorescent staining (IF). HMGB1 expression was increased in PE relative to physiological placentae while RAGE was unvaried. 24 h LMWH treatment significantly up-regulated HMGB1 expression but inhibited HMGB1/RAGE complex formation in physiological explants. RAGE expression decreased in treated relative to untreated explants at 48 h. IF showed HMGB1 localization in both cytoplasm and nucleus of mesenchymal and endothelial cells but not in the trophoblast. IL-6 and TNFα gene expression were significantly increased at 24 h relative to controls, while they were significantly down-regulated in 48 h vs. 24 h LMWH explants. Our data depicted a new molecular mechanism through which LMWH exerts its anti-inflammatory effect on PE placentae, underlying the importance of HMGB1/RAGE axis in PE inflammatory response.

## 1. Introduction

Pre-eclampsia (PE) is a severe placenta-related syndrome that affects 5–8% of all pregnancies, representing one of the main causes of fetal-maternal morbidity and mortality in developed countries [1]. PE is characterized by severe maternal hypertension, proteinuria and exacerbated systemic and placental inflammation [2]. The syndrome becomes evident only after the 20th weeks of gestation but it likely originates during first trimester of pregnancy when the trophoblast invades the maternal decidua causing spiral artery remodeling [1]. In PE, trophoblast invasion is impaired, leading to aberrant placental perfusion, oxidative stress and consequent inflammation and endothelial dysfunction [3,4]. This pathologic environment further promotes the release of pro-inflammatory cytokines and syncithiotrophoblast debris into the maternal circulation, directly activating the endothelium thus causing systemic inflammation [4,5].

Among many players of the PE inflammatory response, there is increasing interest in a group of proteins known as Damage Associated Molecular Patterns (DAMPs) that work as an endogenous danger signal [6,7]. DAMPs have been highlighted as key inflammation activators during PE, preterm labor and pregnancy-related metabolic dysfunction [7]. The High Mobility Group Box 1 (HMGB1) is considered the “prototype” of DAMPs. Discovered 40 years ago as non-histone chromatin associated protein [8], HMGB1 is directly released from activated monocytes and macrophages or passively discharged by damaged/necrotic cells to induce inflammatory cytokines production [9,10], immune cells maturation and tissues remodeling [11,12]. The main mediator of HMGB1 inflammatory activity is the Receptor of Advanced Glycation End products (RAGE) [13]. After HMGB1 binding, RAGE activates the Nuclear Factor kappa B (NF-kB) pathway which induces the production of pro-inflammatory cytokines as Interleukin 6 (IL-6) and Tumor Necrosis Factor α (TNF-α) [14]. IL-6 is pivotal for the acute phase response [15] while TNFα, beside its typical immunomodulatory activities, inhibits first trimester extravillous trophoblast invasion by triggering apoptosis and decreasing cytotrophoblast proliferation [16,17]. Importantly, IL-6 [18,19] and TNFα over-expression were reported in the placental tissue and maternal serum of PE women [16,19,20].

HMGB1 involvement in inflammatory diseases has been widely demonstrated and the administration of anti-HMGB1 antibodies resulted in decreased organ damage and mortality in several models of exacerbated inflammation [15,16,17,18,19]. In PE pathogenesis and placental inflammation, the HMGB1/RAGE role is controversial. In PE placentae, HMGB1 was found increased [20,21] while RAGE expression was reported either significantly higher [22] or unchanged [20].

Despite intensive investigation on PE pathogenesis, no effective therapy is currently available except for a timed and often pre-term delivery. This intervention can resolve maternal symptoms but it could be extremely dangerous for the fetus, causing also severe long-term sequelae. Low Molecular Weight Heparin (LMWH), a renowned anti-coagulant, has been recently investigated as a therapeutic approach for preeclampsia and other pregnancy-related complications. The anticoagulant therapy was first proposed for pregnant women with thrombophilia, characterized by utero-placental thrombosis and poor obstetric outcomes [23]. The use of anticoagulants during pregnancy was suggested more than 40 years ago for women which underwent previous placental infarction [24]. The rationale was that a better placental function might be obtained by reducing the risk of placental thrombosis, thus improving pregnancy outcomes in high-risk subjects [25]. However, heparin seems to not exert an anticoagulant effect on the placental tissue since its administration does not improve placental thrombotic injury rate [26]. Interestingly, heparin improves pregnancy outcomes with a decrease of placental-mediated complications also in women without thrombophilia [25,27]. In a recent systematic review and meta-analysis, Rodger and colleagues reported that LMWH reduces the risk of preeclampsia recurrence, indicating LMWH as a promising therapeutic tool for PE syndrome [28]. However, heparin use for preeclampsia treatment is still controversial and its placental mechanism of action is unclear. It has been proposed that the beneficial effects of LMWH on PE patients are correlated to an anti-inflammatory action. Heparin and its derivatives have been demonstrated to be effective against several inflammatory experimental conditions as mouse models of colitis [29], arthritis [30] and in patient with airways inflammatory disease [31]. Furthermore, it has been shown that, in an inflammation-model of mice treated with LPS, modified heparin with low/absent anticoagulatory capacity strongly inhibited expression of hepcidin, serum iron uptake regulator whose levels rise during inflammation [32].

Importantly, it has been demonstrated in vitro that heparin reduces HMGB1 affinity for RAGE [33,34]. HMGB1 possesses heparin-binding ability [35] and it undergoes conformational change when mixed with LMWH [34]. Moreover, HMGB1-stimulated macrophages and Human Umbilical Vein Endothelial Cells (HUVEC) reduce IL-6 and TNF-α release when treated by heparin in a dose-dependent manner [34].

Our hypothesis is that LMWH inhibition of the placental pro-inflammatory HMGB1/RAGE axis could contribute to the anti-inflammatory activity observed in heparin-treated pre-eclamptic patients. In the present study, we evaluated placental HMGB1 and RAGE expression in physiological and PE pregnancies and we investigated whether LMWH could modify placental HMGB1 and RAGE expression and/or their binding ability in the human placental tissue.

## 2. Results

### 2.1. Study Population

Clinical features of the study population are reported in Table 1. Physiological (n = 19) and PE (n = 18) patients were comparable in terms of maternal age and parity. As expected, PE group was characterized by significantly lower gestational age (*p* < 0.001), birth weight (*p* < 0.01) and placental weight at delivery (*p* < 0.001) relative to control pregnancies. 

The presence of fetal-placental compromise was evaluated by Doppler velocimetry of umbilical and uterine arteries. PE patients had abnormal Doppler velocimetries of umbilical or uterine (50% and 66.67% respectively) arteries, while in eight PE patients (44.44%) both values were pathological. In our study population, LMWH was given during pregnancy to one patient of the control group (5.26%) and to three patients of the PE group (16.66%).

### 2.2. HMGB1 and RAGE Expressions in Physiological and Pre-Eclamptic Placentae

HMGB1 has been widely studied for its ability in stimulating pro-inflammatory cytokines by binding to its receptor RAGE. Since pre-eclampsia is characterized by exacerbated placental inflammation, we investigated HMGB1 and RAGE expression in PE compared to physiological placentae.

**Figure 1:**
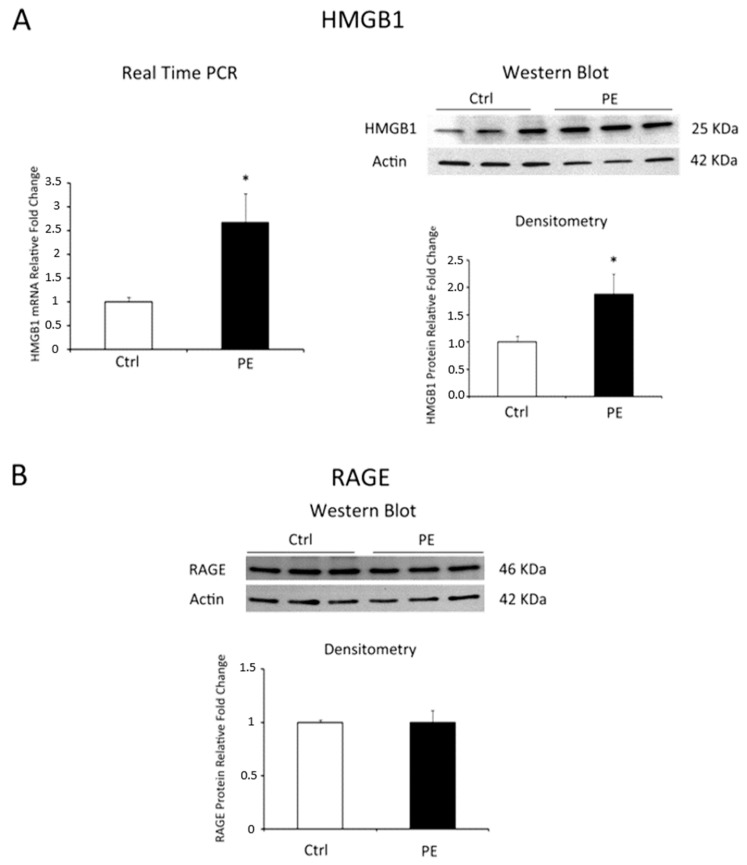
HMGB1 and RAGE expression levels in normal (N = 19) versus PE (N = 18) placentae. (**A**) HMGB1 mRNA (left panel) and protein (right panel) expression levels as assessed by Real Time PCR and western blot analysis; (**B**) RAGE protein expression assessed by western blot. Statistical significance (*) has been considered as *p* < 0.05.

We reported a significant increase of HMGB1 mRNA levels in PE vs. physiological placentae (*p* = 0.018, 2.67 fold increase, Figure 1A, left panel). Data were confirmed at the protein level where we found a 1.76 Fold Increase in PE placentae relative to controls (*p* = 0.001, Figure 1A, right panel). Western blot analysis for RAGE did not reveal difference in its protein expression between pathological and control tissues (Figure 1B).

Since PE is often associated with Fetal Growth Restriction (FGR) [36,37], we investigated whether HMBG1 expression in PE placentae could be related to fetal growth. We found no significant differences in placental HMGB1 expression between PE with Appropriate for Gestational Age (AGA) fetuses PE-FGR placentae (*p* > 0.05, Figure S1A). Importantly, PE-AGA and PE-FGR placentae were both characterized by higher gene (PE-AGA vs. Ctrl, *p* = 0.296, 3.29 Fold Increase; PE-FGR vs. Ctrl, *p* = 0.019, 2.41 Fold Increase, Figure S1A) and protein (PE-AGA vs. Ctrl, *p* = 0.028, 1.72 Fold Increase; PE-FGR vs. Ctrl, *p* = 0.02, 1.77 Fold Increase, Figure S1B) HMGB1 expression levels relative to physiological controls.

Next, we compared HMGB1 levels in early-onset versus late-onset PE in order to highlight a possible relation between HMGB1 expression and gestational age during preeclampsia. We did not find significant differences in HMGB1 gene and protein expression levels between early- and late-onset PE (*p* > 0.05, Figure S2A,B).

Finally, since labour is a pro-inflammatory condition [38], we investigated whether HMGB1 expression was affected by the onset of labour. No significant differences were found in HMGB1 mRNA and protein levels in placentae from patients in labour relative to those that underwent caesarean section (CS) in both control and PE groups (*p* > 0.05, Figure S3A,B).

### 2.3. LMWH Effect on HMGB1 and RAGE Expression in Physiological Human Villous Explants

**Figure 2:**
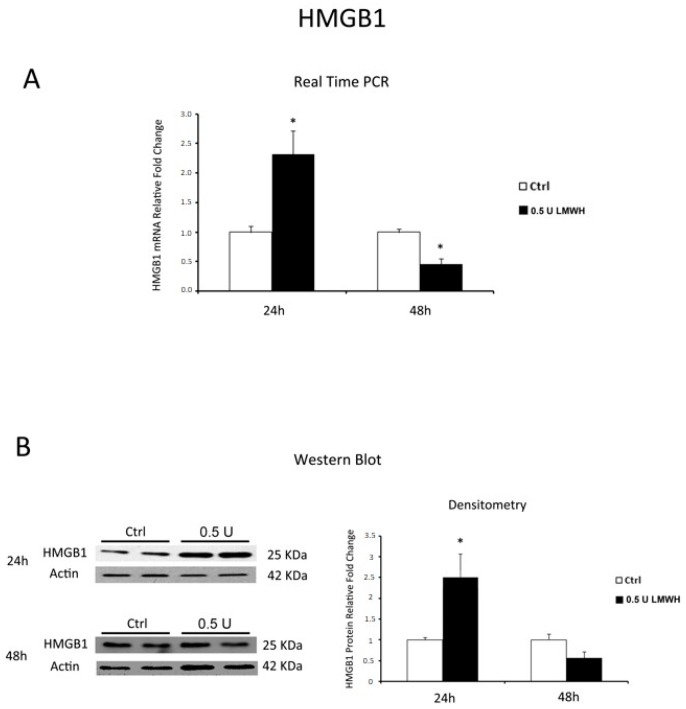
HMGB1 expression in LMWH-treated vs. untreated physiological placental villous explants. (**A**) HMGB1 mRNA expression levels in placental villous explants treated for 24 h (ctrl n = 8 and 0.5 U LMWH n = 10) and 48 h (ctrl n = 11 and 0.5 U LMWH n = 10) by 0.5 U of LMWH or plain culture medium, assessed by Real Time PCR; (**B**) Representative western blot for HMGB1 protein expression in LMWH-treated vs. untreated placental villous explants for 24 h (ctrl n = 4 and 0.5 U LMWH n = 4) and 48 h (ctrl n = 10 and 0.5 U LMWH n = 10). Statistical significance (*) has been considered as *p* < 0.05.

Low Molecular Weight Heparin, widely known for its anti-coagulant properties, has beneficial effects on PE patients that look correlated to an unspecified anti-inflammatory activity. Therefore, we next investigated whether LMWH could modulate placental HMGB1-mediated inflammation in physiological tissues. Since recent evidences demonstrated that LMWH dose-response is highly variable [39], we tested LMWH effects at 24 and 48 h using concentrations of 0.5 and 5 units/mL to span the range of maternal circulating plasma levels found in pregnant women receiving subcutaneous injections of prophylactic (5000 units/day; equivalent to 0.25 units/mL) or therapeutic (more than 10,000 units/day; equivalent to 2.5 units/mL) doses [40,41]. 5 U of LMWH resulted toxic to our model as we reported, as a sign of tissue degradation, an abnormally low expression of housekeeping gene 18S (data not shown). Therefore, we considered only data obtained from 0.5 U LMWH-treated explants. After 24 h, we found significantly increased HMGB1 gene expression in treated compared to control explants (*p* = 0.001, 2.32 fold increase, Figure 2A). In contrast, we found a significant decrease of HMGB1 gene expression in 48 h in LMWH-treated explants relative to controls (*p* = 0.036, 2.22 fold decrease, Figure 2A). Data were confirmed at the protein level by western blot analysis. We found a significant increase in HMGB1 protein levels (*p* = 0.029, 2.5 fold increase, Figure 2B) at 24 h that markedly decreased at 48 h in LMWH explants versus controls (*p* > 0.05, 1.78 fold decrease, Figure 2B).

**Figure 3:**
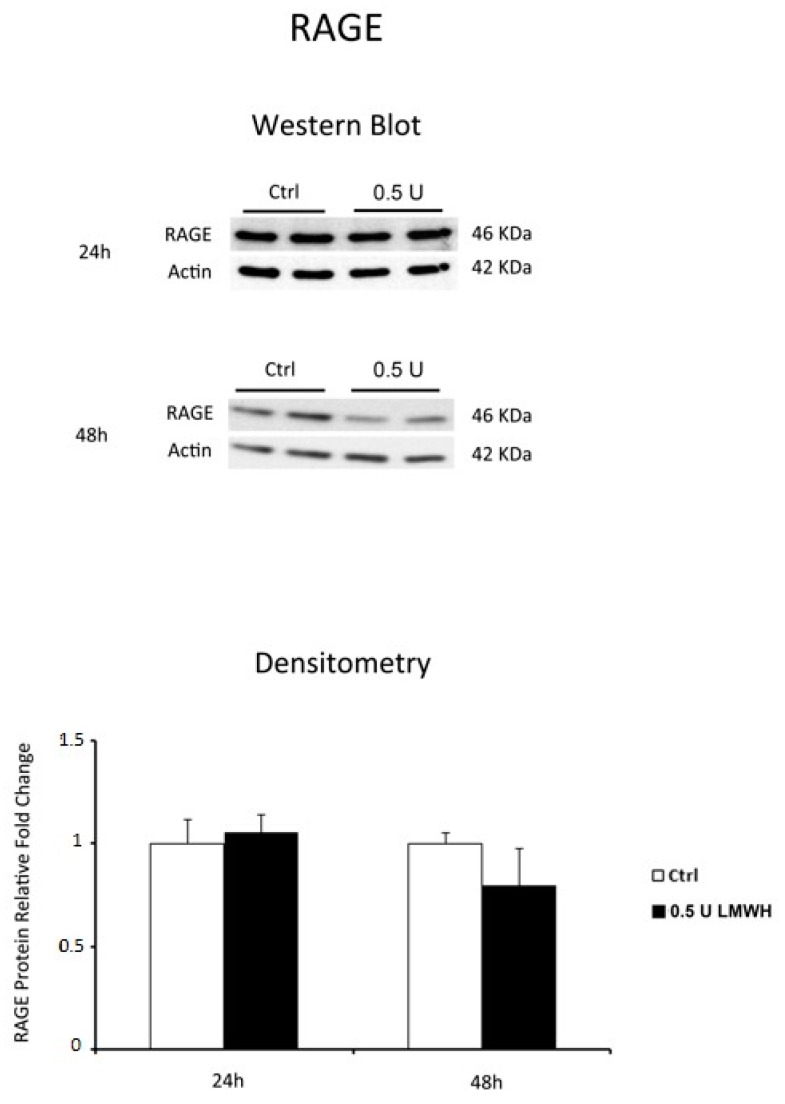
RAGE expression in LMWH-treated vs. untreated physiological placental villous explants. Representative western blot for RAGE protein expression level in physiological term placental villous explants treated for 24 h (ctrl n = 7 and 0.5 U LMWH n = 7) and 48 h (ctrl n = 10 and 0.5 U LMWH n = 10) by 0.5 U of LMWH.

Finally, we investigated whether LMWH could modulate RAGE expression. Western blot data showed no significant differences in RAGE protein levels between LMWH-treated and untreated explants at 24 h, while RAGE protein expression was significantly inhibited at 48 h after LMWH treatment (*p* = 0.014, 1.85 Fold decrease) (Figure 3). Our findings suggest the ability of LMWH to modulate both HMGB1 and RAGE expression in term human placental villous explants.

### 2.4. 0.5 U LMWH Treatment Did Not Affect Physiological Term Placental Villous Explants Viability

To evaluate whether 0.5 U LMWH affects placental explants viability, we performed the Lactate Dehydrogenase (LDH) assay. This test determines cell membrane integrity by measuring cellular LDH release/leakage. After 24 h and 48 h of treatment, we observed that the relative LDH amount released into the media was comparable among basal culture medium treated explants and those treated by 0.5 U LMWH (*p* > 0.05, Figure S4). Importantly, LDH levels in all treated explants were significantly lower compare with the cytotoxicity cut-off value of 41% ranging from 0.13 to 0.56% LDH release (*p* < 0.05, Figure S4). Therefore, 0.5 U LMWH treatment does not affect cell viability (*p* < 0.05, Figure S4).

### 2.5. LMWH Inhibits HMGB1/RAGE Interaction

**Figure 4:**
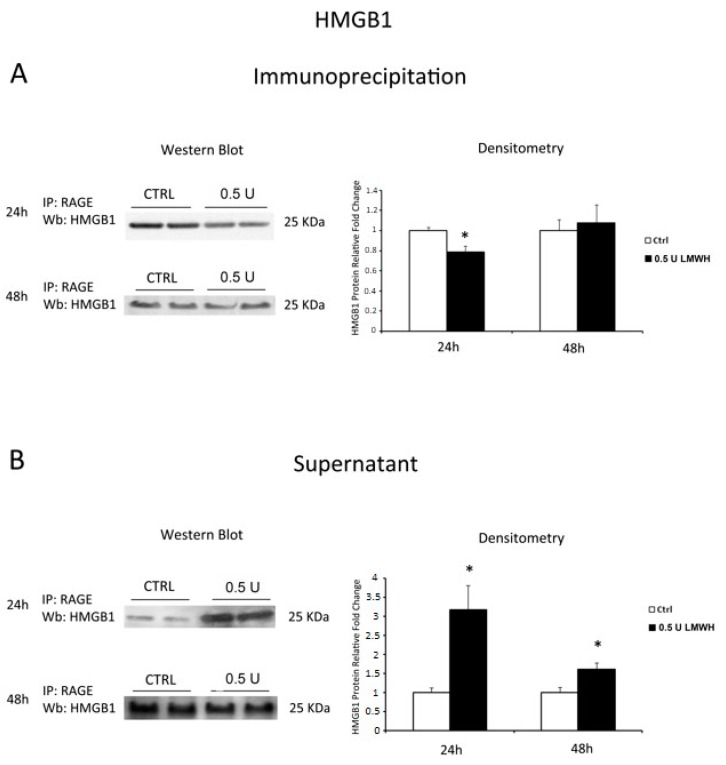
RAGE/HMGB1 binding in LMWH-treated vs. untreated placental villous explants. HMGB1 protein levels were evaluated by western blot after immunoprecipitation with RAGE antibody of protein lysates from placental villous explants treated by 0.5 U of LMWH for 24 h and 48 h. (**A**) Representative WB for HMGB1 protein levels after RAGE IP at 24 h (ctrl n = 16 and 0.5 U LMWH n = 15) and 48 h (ctrl n = 5 and 0.5 U LMWH n = 6); (**B**) Representative WB for HMGB1 protein levels in RAGE IP supernatant at 24 h (ctrl n = 8 and 0.5 U LMWH n = 10) and 48 h (ctrl n = 5 and 0.5 U LMWH n = 6). Statistical significance (*) has been considered as *p* < 0.05.

HMGB1 pro-inflammatory effect is mediated by its interaction with RAGE. Indeed, we investigated the ability of HMGB1 to bind its receptor after 24/48 h LMWH treatments. To reach our goal we performed RAGE immunoprecipitation followed by HMGB1 western blot. We found a significant decrease in HMGB1 protein levels (*p* = 0.011, 1.27 fold decrease, Figure 4A) in 24 h LMWH-treated explants. In contrast, we found no differences of HMGB1 relative to untreated controls in 48 h-treated villous explants (Figure 4A). To further confirm our findings, we also determined HMGB1 protein levels in the IP supernatants. Western blot analysis revealed a significant increase of free HMGB1 in the supernatants at both 24–48 h LMWH-treated explants compared to untreated controls (*p* < 0.001 and *p* = 0.017 respectively, 3.17 and 1.61 fold increases, respectively, Figure 4B).

### 2.6. HMGB1 Immunofluorescent Staining in Physiological Placental Villous Explants

**Figure 5:**
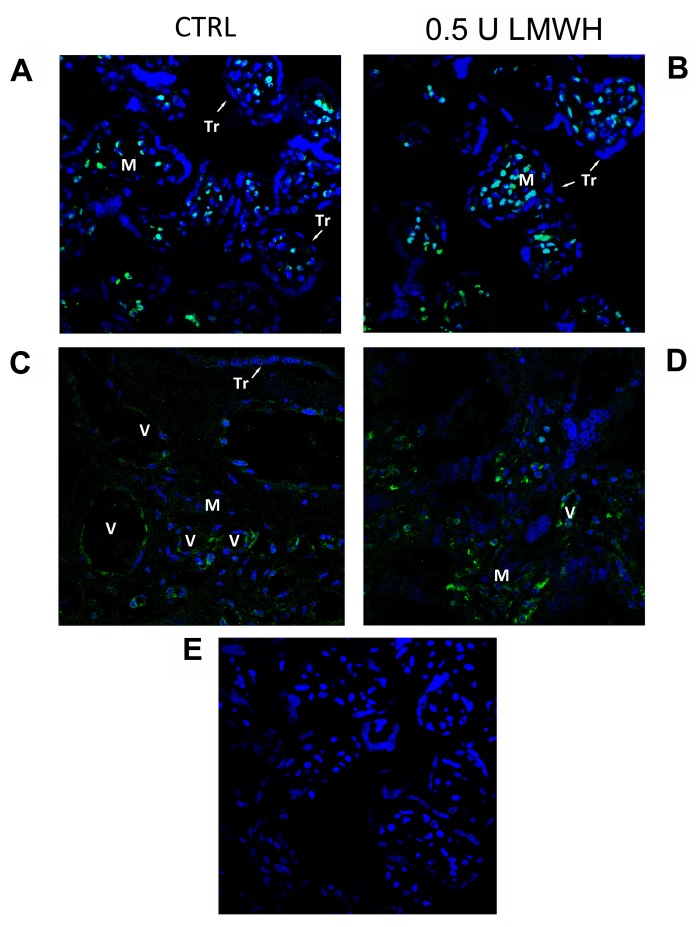
HMGB1 spatial localization in untreated or 0.5 U LMWH treated physiological villous explants assessed by immunofluorescent staining. (**A**–**C**) HMGB1 spatial localization in untreated (n = 6) physiological villous explants assessed by immunofluorescent staining; (**B**–**D**) HMGB1 spatial localization in physiological villous explants treated with 0.5 U of LMWH (n = 6); (**E**) Absence of positive immunoreactivity for HMGB1 in section stained with control IgG. Cell nuclei are showed in blue by DAPI signal. TR, trophoblast cells; M, mesenchyme; V, vessel. Original magnifications, 60×.

Next, to elucidate which villous cell population was most responsive to LMWH treatment in terms of HMGB1 expression, we performed HMGB1 immunofluorescence in 24 h treated and control explants. HMGB1 operates as transcription factor when it is localized in the nucleus, while it acts as a pro-inflammatory cytokine when released from the cytoplasm to the extracellular environment. HMGB1 IF staining was mainly localized in mesenchymal (M) and endothelial cells and absent in the syncytiotrophoblast (TR) layer (Figure 5A–C). Importantly, IF confirmed RNA and protein data at 24 h of LMWH treatment. Immunoreactivity for HMGB1 was markedly increased in mesenchymal and endothelial cells nuclear, peri-nuclear and cytoplasmic areas in LMWH-treated explants (Figure 5B–D) relative to untreated controls (Figure 5A–C).

### 2.7. IL-6 and TNFα Gene Expression in Physiological Placental Villous Explants

**Figure 6:**
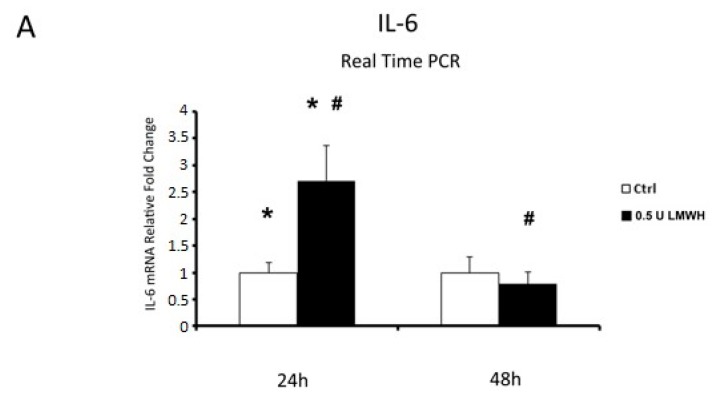

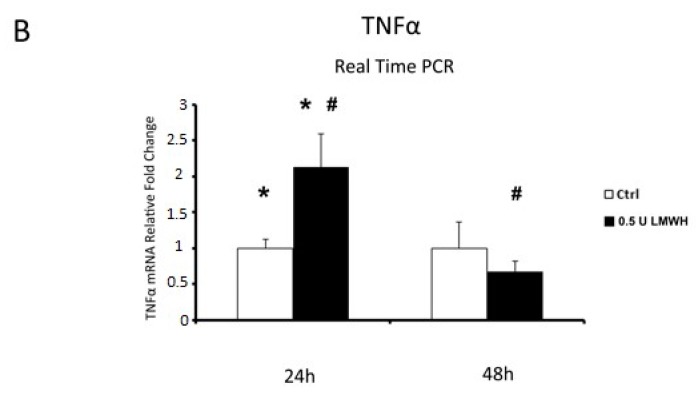
IL-6 and TNFα mRNA expression in LMWH-treated explants vs. controls assessed by Real Time PCR. (**A**) IL-6 gene expression in villous explants treated by 0.5 U of LMWH for 24 h (ctrl n = 8 and 0.5 U LMWH n = 10) and 48 h (ctrl n = 11 and 0.5 U LMWH n = 10). (**B**) TNFα gene expression levels in villous explants treated by 0.5 U of LMWH for 24 h (ctrl n = 8 and 0.5 U LMWH n = 10) and 48 h (ctrl n = 11 and 0.5 U LMWH n = 10). Statistical significance (*#) has been considered as *p* < 0.05.

Finally, since we established that LMWH could impair HMGB1/RAGE binding, we examined by Real Time PCR the expression of HMGB1 target genes IL-6 and TNFα in placental villous explants treated by 0.5 U LMWH or basal culture medium for 24 or 48 h. We focused on gene expression to emphasize HMGB1 role as transcriptional activator. Accordingly to HMGB1 expression, we found significantly higher IL-6 (*p* = 0.04, 2.7 fold increase, Figure 6A) and TNFα (*p* = 0.02, 2.13 fold increase, Figure 6B) mRNA levels at 24 h in LMWH-treated explants vs. controls. No significant differences were found in IL-6 and TNFα gene expression levels in 48 h LMWH-treated explants vs. controls (Figure 6A,B), even though it was evident a trend of reduction in both genes after LMWH administration. Importantly, considering LMWH treated explants, we found significantly lower IL-6 (*p* = 0.037, 3.4 fold decrease, Figure 6A) and TNFα (*p* = 0.02, 2.7 fold increase, Figure 6B) mRNA levels in 48 h vs. 24 h group.

## 3. Discussion

In the present study, we reported increased expression of HMGB1, a potent inducer of pro-inflammatory cytokines, in placentae from pregnancies complicated by pre-eclampsia compared to physiological controls. We did not find differences in the expression of HMGB1’s receptor RAGE in PE tissues relative to controls. Next, we evaluated LMWH effect on HMGB1/RAGE expression and association as well as on HMGB1 target genes IL-6 and TNFα in a physiological placental villous explants model. After 24 h, LMWH promoted HMGB1, IL-6 and TNFα while it did not influence RAGE expression. Importantly, HMGB1/RAGE binding was inhibited by LMWH at 24 h, as further demonstrated by the accumulation of free HMGB1 in treated explants. Finally, HMGB1, RAGE, IL-6 and TNFα were down-regulated at 48 h after 0.5 U LMWH treatment. Our data indicate that LMWH modulates the HMGB1/RAGE pro-inflammatory axis in the human placenta.

HMGB1 placental expression was first reported in 2007 by Holmlund and colleagues, that described its localization in both syncithiotrophoblast and chorionic mesenchyme [20]. Herein we reported a significant over-expression of HMGB1 in PE placentae, characterized by exacerbated inflammation. In accordance to our results, Wang and colleagues previously reported increased HMGB1 levels in the placentae, but not in the serum, of pre-eclamptic patients [20,21]. Importantly, they found no association between gestational age and HMGB1 serum concentration [21], hypothesizing that the lack of correlation between placental and systemic HMGB1 levels could be due to a source of circulating HMGB1 different from the placenta and/or to the elevated serum levels of soluble RAGE (sRAGE) in PE [21,42]. Their PE patients were selected by means of systolic/diastolic blood pressure >140/90 mmHg and proteinuria >0.3 g/24 h and no stratification of severe cases was made. In contrast, Holmlund and colleagues included severe cases (systolic/diastolic blood pressure >160/110 and proteinuria >3 g/24 h) but they did not find a correlation with HMGB1 placental expression and PE severity [20].

The elevated HMGB1 levels that we described in the pre-eclamptic placental tissue could contribute to the pro-inflammatory cytokines production and/or they could be a consequence of the placental ischemic damage typical of PE. Recently, it was reported that serum HMGB1 levels are increased in pregnant relative to non-pregnant women and that this increase is more dramatic in PE, without differences between moderate and severe pre-eclampsia [43]. In line with these data, herein we demonstrated that placental HMGB1 increase during pre-eclampsia is not related to the presence of fetal growth restriction. In accordance to our results, Chen and colleagues reported no significant difference in HMGB1 levels between PE-AGA and PE-FGR placentae [44]. Taken together, all these findings suggest the presence of a common pathogenesis between PE-AGA and PE-FGR pregnancies.

We did not find any association between PE onset (early or late) and HMGB1 expression levels. Our data are in agreement with Chen and colleagues that, using semi-quantitative analysis of the immunohistochemistry and western blotting, showed no significant differences in the levels of HMGB1 in the syncytiotrophoblast from early-onset relative to late-onset PE and control pregnancies [44]. Importantly, in the present study we focused only on the last trimester of pregnancy since it is correlated to the onset of PE clinical symptoms. Our future investigation will be consider HMGB1 expression throughout gestation.

Elevated HMGB1 was observed in pregnant women with other pro-inflammatory conditions as obesity [45] and pre-term labor [46]. It is well established that labour is associated to a pro-inflammatory systemic response [38]. Since HMGB1 is a potent inflammatory cytokine, we investigated placental HMGB1 expression in labour versus caesarean section deliveries, but no differences were observed in both control and PE groups. In line with our results, Holmlund and colleagues described no significant differences in HMGB1, RAGE, TLR2 or TLR4 expression between labour and CS placentae [20].

Thus, different stress stimuli, not only of placental origin, could be responsible for the elevated HMGB1 levels observed. It was shown that JEG-3 choriocarcinoma cells expressed and released large amount of HMGB1 under PE-like hypoxic conditions [47], suggesting a possible involvement of placental HMGB1 in the instauration of the PE exacerbated inflammation [48,49].

Extracellular HMGB1 exerts its cytokine-like activity by binding to RAGE receptor. RAGE increases with pregnancy onset and it is further augmented in the PE myometrium [22]. Moreover, RAGE protein expression was found significantly higher in syncithiotrophoblast and endothelial cells of PE placentae relative to controls [50]. In stark contrast, herein we did not find differences in RAGE expression between PE and control placentae. In accordance with our findings, other groups found abundant RAGE expression in trophoblast and decidua of normal term placentae but no differences in PE versus normal tissues [20]. Variable results could be due to differences in the specimens used. We analyzed the whole placental tissue instead of isolated trophoblast or endothelial cells.

Several evidences indicate beneficial effects of LMWH for PE treatment. LMWH seems to improve the maternal-fetal outcomes [27] and to reduce pregnancy-complications recurrence [28]. Moreover, LMWH effects are unrelated to the heparin anti-coagulatory activity [26,27]. It has been shown in vitro that conditioned media from heparin-treated placental villi exerted a pro-angiogenic activity on Human Umbilical Vein Endothelial Cells (HUVECs) [40] and that LMWH promotes trophoblast turnover by stimulating cytotrophoblast proliferation and syncytial fusion [41]. However, Drewlo and colleagues demonstrated that LMWH could stimulate the placental expression and release of anti-angiogenic soluble Vascular Endothelial Growth Factor (VEGF) receptor-1 (sFLT) and thus they recommended caution for LMWH use during severe PE [41].

Indeed, LMWH use is controversial and its mechanism of action on the placental tissues is still unclear. In vitro studies suggested LMWH ability to reduce trophoblast apoptosis through the modulation of anti-apoptotic B-cell lymphoma (Bcl-2) family molecules [51] and to promote the invasiveness of extravillous trophoblast by enhancing Matrix metalloprotease-2 (MMP-2), heparin binding-Epidermal Growth Factor (HB-EGF) and cysteine-rich angiogenic inducer 61 (Cyr61) activities [52]. Herein, we reported LMWH ability to modulate the pro-inflammatory HMGB1/RAGE axis in physiological term placental tissues. We found that HMGB1/RAGE interaction was inhibited by LMWH after 24 h of treatment as demonstrated by immunoprecipitation assay. This effect could be due to the LMWH-induced HMGB1 conformational change previously demonstrated in vitro [34]. Since RAGE binds heparin too [33], we could not exclude that LMWH could have blocked also the HMGB1 receptor.

Furthermore, HMGB1/RAGE complex could modulate NFkB activation that, in turn, promotes RAGE expression [53]. Thus, RAGE reduction reported herein at 48 h of treatment could be directly due to LMWH-mediated inhibition of HMGB1/RAGE complex. The initiation of a pro-inflammatory loop by HMGB1 has been suggested in several models of pathological inflammation as multiple sclerosis [54], necrosis [55] and preterm birth [56]. In PE, LMWH might be able to slow or even stop the HMGB1-induced pro-inflammatory loop preventing the exacerbation of inflammation typical of this syndrome.

Taken together, our data confirm the ability of low molecular weight heparin to block the HMGB1/RAGE interaction in vitro in placental villous explants. One limitation of the present study is that we did not experimentally confirm the LMWH-induced HMGB1 conformational change. Moreover, HMGB1 possesses other receptors like the Toll Like Receptor (TLR) 2 and TLR4 that lead to NFkB nuclear translocation as well [57,58]. It has been recently demonstrated that 2-O, 3-O-desulfated heparin, characterized by significant anti-inflammatory but minimal anti-coagulatory properties, is able to inhibit HMGB1 interaction with TLR2 and TLR4 [59]. Indeed, TLR2 and TLR4 could have contributed to the heparin-driven modifications reported in the present study. Further investigations are required.

Since its discovery in the late 1990s, HMGB1 was mainly considered as a nuclear protein but evidences were accumulating about its extracellular cytokine-like function. Similar to other cytokines, HMGB1 is pleiotropic with cell-specific activities in the placental tissue [20]. Previous data showed that HMGB1 induced migration and sprouting in endothelial cell and promoted trophoblast invasiveness [60,61]. Zhu and colleagues reported HMGB1 cytoplasmatic localization in the PE placenta while in physiological term pregnancies it was described in the placental nuclei [62]. These data suggest that nuclear-cytoplasmatic translocation may participate in the pathogenetic process of Preeclampsia. In line with these results, we found a stronger nuclear/perinuclear HMGB1 signal relative to the cytoplasm. In all LMWH-treated and untreated physiological term placental explants. Moreover, we showed that HMGB1 signal was mainly localized in the chorionic mesenchyme and endothelial cells. Our findings are in accordance with Holmlund and colleagues that found HMGB1 nuclear expression in both chorionic mesenchyme and endothelial cells [20,60]. In contrast, previous studies described a strong HMGB1 positivity in both syncytiotrophoblast and extravillous trophoblast [20,62].

The activation of HMGB1/RAGE pathway promotes the transcription of IL-6 and TNF-α [34,53]. In our model, the inhibition of IL-6 and TNF-α expression was evident after 48 h of LMWH-treatment. Taken together, our data confirmed the ability of low molecular weight heparin to block HMGB1/RAGE interaction and the expression of their target genes in an in vitro model of placental villous explants. The increase in HMGB1, IL6 and TNFα expression at 24 h in response to LMWH suggest an initial inflammatory stress being counteracted by heparin as demonstrated by the inhibition of HMGB1/RAGE binding reported in the present study. It is well established that heparin could directly modify HMGB1 structure altering its interaction with RAGE, that in turn leads to a decrease of IL-6 and TNF-α expression [34]. Importantly, HMGB1 transcription itself is stimulated by pro-inflammatory TNF-α [63] as well as TNF-α could promote HMGB1 release from inflammatory cells [14,64]. Thus, the inhibition of TNF-α production exerted by LMWH could have indirectly contributed to HMGB1 down-regulation that we reported.

In conclusion, our data depicted a new molecular mechanism through which LMWH exerts its anti-inflammatory activity on the placental tissue. We underlined the importance of the HMGB1/RAGE axis in the placental inflammatory response thus opening new directions in the investigation of PE pathogenesis and management. Indeed, targeting HMGB1 could be effective to control the ischemia-related inflammatory damage typical of this syndrome, as already demonstrated in other pathological models [65,66,67]. Further investigation is required.

## 4. Materials and Methods 

### 4.1. Ethics Statement and Place of Recruitment

This study was conducted according to the principles expressed in the Declaration of Helsinki. The study was approved by the Institutional Review Board of O.I.R.M. S. Anna Hospital and ‘‘Ordine Mauriziano di Torino’’ (n 337; protocol 11552/C.28.1 07/12/2010) (Turin, Italy). All patients were recruited at O.I.R.M S. Anna Hospital (Turin, Italy) and provided written informed consent for the collection of samples and subsequent analysis.

### 4.2. Study Population and Tissues Collection

The study populations included pre-eclamptic singleton pregnancies (n = 18) and physiological control term pregnancies (n = 19). PE was defined by presence of pregnancy-induced hypertension (systolic >140 mmHg, diastolic >90 mmHg) and proteinuria (>300 mg/24 h) after the 20th weeks of gestation in previously normotensive women [1]. Fetal Growth Restriction (FGR) was defined as birth weight below the fifth centile according to the Italian growth curves normalized for gestational age and sex [36,37] accompanied by pathological umbilical artery Doppler waveforms (absent or reverse end diastolic flow—A/REDF) and/or increased resistance to flow in maternal uterine arteries (early diastolic notch or pulsatility index—PI—more than 0.58). Early-onset PE was defined as preeclampsia that develops before 34 weeks of gestation, whereas late-onset preeclampsia develops at or after 34 weeks of gestation [68]. Physiological controls were obtained from normal term healthy singleton pregnancies that did not show any signs of preeclampsia or other placental disease. We did not use “age-matched” control pregnancies since no pre-term delivery is physiological. Patients with cardiovascular diseases, diabetes, metabolic syndrome, infections, kidney disease, congenital malformations and chromosomal anomalies (number and/or structure) were excluded. Placental tissue biopsies were randomly collected from the basal plate and snap frozen immediately after delivery. Calcified, necrotic and visually ischemic areas were excluded from collection. Eight additional physiological term placentae were collected and processed for placental villous explants preparation as described below.

### 4.3. Human Chorionic Villous Explants Cultures and LMWH Treatment

Biopsies from physiological term placentae (n = 8) were processed within 2 h from delivery. This model allowed us to determine the sequence of molecular events in healthy tissues characterized by conserved physiological pathways, thus avoiding biases due to previous existing pathological anomalies. Amniotic membranes were mechanically removed, and placental specimens were washed in cold phosphate-buffered saline (PBS) solution to eliminate excess of blood. Small portions of placental chorionic villi were excised and placed in a 24-well culture dishes (35 mg, n = 142). Explants were cultured in Ham’s F12 media (Gibco by Life Technologies, Monza, Italy) and incubated at 37 °C and 5% CO_2_ to equilibrate overnight. Explants were then removed from the culture media and placed in 500 μL of medium with different LMWH (Parnaparin, Alfa Wassermann, Bologna, Italy) concentrations: 0.5 U and 5 U. Explants in basal culture medium were used as controls. Finally, control and treated explants were collected after 24 h or 48 h and immediately frozen for RNA and protein isolation (n = 130) or fixed for histological analyses (n = 12).

### 4.4. Lactate Dehydrogenase (LDH) Cytotoxicity Assay

Cytotoxicity of 0.5 U LMWH treatment on villous explants was determined by LDH-Cytotoxicity colorimetric assay (Cat. No. K726-500, Biovision Inc., Milpitas, CA, USA) performed using 24 h and 48 h explants supernatant according to the manufacturer’s instructions. Explants treated by Triton X-100 and by basal culture medium for 8 h were used as high (H, 100%) and low controls (0%) respectively. The positive control (+) was provided by the manufacturer to test whether all reagents were working properly responding to active LDH enzyme. The absorbance at 450 nm was measured using a plate reader spectrophotomether. Percent cytotoxicity values were determined based on the amount of LDH released from 30-min readings, as follows: (test sample − low control)/(high control − low control) × 100. The cut-off values for the LDH assay was determined by the 2 × SD rule where the threshold is defined as 2 × SD beyond the mean of the screened samples. Values equal or exceeding the threshold were considered as sign of cytotoxicity.

### 4.5. RNA Isolation and Real Time PCR

Total RNA was extracted from frozen placental biopsies and explants using TRI reagent (Sigma-Aldrich, Milano, Italy) according to manufacturer instructions and next treated with DNAse I to remove genomic DNA contamination. Three µg of total RNA were reverse transcribed using a random hexamers approach (Fermentas Europe, St. Leon-Rot., Germany). Gene expression levels of HMGB1, IL-6 and TNFα were quantified by Real-time PCR using specific TaqMan primers and probes following manufacturer’s protocol (Life Technologies). For the relative quantitation, PCR signals were compared among groups after normalization using ribosomal 18S RNA expression as internal reference (Life Technologies). 18S mRNA expression was not affected by LMWH treatment. Relative expression and fold change were calculated according to Livak and Schmittgen [69].

### 4.6. Western Blot Analysis

Total proteins were isolated from placental biopsies and villous explants using 1X Radio Immuno-precipitation Assay (RIPA) buffer supplemented with Protease Inhibitors. Thirty µg of protein were processed by SDS-page electrophoresis on 4–20% polyacrylamide pre-cast gradient gels (Bio-Rad Laboratories S.r.l., Segrate, Italy). Next, proteins were transferred on polyvinylidene fluoride (PVDF) membranes and probed at room temperature with primary antibodies using the SnapID 2.0 system (Merck-Millipore, Vimodrone, Italy) following manufacturer instructions. The following primary antibodies were used: mouse monoclonal anti-human HMGB1 (1:1500 dilution, Sigma-Aldrich, Milano, Italy) and anti-human RAGE (1:500 dilution, Santa Cruz Biotechnology, D.B.A. Italia S.R.L, Segrate Italy). Biotinylated secondary antibodies were goat anti-mouse for HMGB1 and RAGE (1:20.000 dilution and 1:2000 dilution respectively, Santa Cruz Biotechnology, D.B.A. Italia S.R.L, Segrate Italy). Protein expression levels were normalized to β-actin by blotting with mouse monoclonal anti-human β-actin antibody (1:1000, Sigma–Aldrich, Milano, Italy). Blots were visualized using “Luminata Classico” Western HRP substrate (Merck-Millipore, Vimodrone Italy) for HMGB1 according to manufacturer’s instructions and followed by densitometry.

### 4.7. HMGB1 Immunofluorescence (IF) Staining

0.5 U LMWH-treated (n = 6) and untreated (n = 6) villous explants were fixed in 4% paraformaldehyde and embedded in paraffin. Sodium citrate antigen retrieval was performed, followed by Sudan Black treatment (0.1% Sudan Black in 70% EtOH) to quench endogenous fluorescence. Sections were pre-incubated in 5% horse serum in PBS (contained 0.04% sodium azide and 0.008% gelatin) to block nonspecific binding and incubated with primary antibodies overnight at 4 °C. Mouse polyclonal antibodies anti-human HMGB1 (1:1000 dilution; Sigma-Aldrich, Milano, Italy) was used. Control IgG were used as negative controls. Slides were treated with 0.4% DAPI (4′,6-diamidino-2-phenylindole) for nuclear detection. IF images were captured using Confocal Nikon D-Eclipse C1 microscope (Nikon, Chiyoda, Tokyo, Japan).

### 4.8. Immunoprecipitation (IP) Assay

Five hundred µg of total proteins from LMWH-treated and control villous explants were incubated with 2 µg of anti-RAGE antibody for two hours at 4 °C on a rotor. Next, 20 µL of G-PLUS-Agarose beads (Santa Cruz Biotechnology, D.B.A. Italia S.R.L., Segrate, Italy) were added to each sample before incubation overnight at 4 °C on a rotor. Samples were then centrifuged at 2500 rpm for 5 min at 4 °C and the supernatants were removed. Pellets were then washed once with 500 µL of PBS and next two times using 500 µL of RIPA buffer plus protease inhibitors. Immunoprecipitates and supernatants were finally processed by western blot analysis for HMGB1 as reported above.

### 4.9. Statistical Analysis

Clinical data are presented as median, range and frequencies (percentages) while in figures as mean and standard error (SE). To compare continuous variables, we used the Mann-Whitney U Test while for categorical variables the comparison between the 2 groups was done with χ^2^-test. Statistical test were carried out using the SPSS statistics version 20 software (IBM Inc., Armonk, NY, USA) and significance was accepted at *p* < 0.05.

## Figures and Tables

**Table 1:** Clinical features of the study population. Values are expressed as median (range) and percentage. A.G.A, appropriate for gestational age; FGR, fetal growth restriction; n.s. not significant.

	Physiological (n = 19)	Preeclampsia (n = 18)	*p* Value
Nulliparae (%)	47.36	50	n.s.
Gestational age at delivery (weeks)	39.4 (38–41)	32.8 (28–37)	<0.001
Maternal age at delivery (years)	34 (23–39)	34 (20–41)	n.s.
Ethnicity (%)			
Caucasian	89.47	86.33	n.s.
Prenatal medications (%)			
Albumin	-	11.11	n.s.
Antibiotics	42.1	16.67	n.s.
Antidepressant	-	-	-
Diuretics	-	16.67	n.s.
Eutirox	5.26	-	n.s.
Folin	84.21	94.44	n.s.
Heparin	5.26	16.66	n.s.
Iron	15.79	5.55	n.s.
Proton Pump Inhibitors (PPIs)	5.26	11.11	n.s.
Ventolin	-	-	-
Smokers (%)	15.78	16.67	n.s.
Alcohol (%)	10.52	16.67	n.s.
Previous prenatal admission (%)	5.26	16.67	n.s.
Systolic Blood pressure (mm Hg)	120 (90–140)	162.5 (130–180)	<0.001
Diastolic Blood pressure (mm Hg)	75 (60–90)	100 (85–117)	<0.001
Proteinuria (g/24 h)	Absent	1.2 (0.33–8.28)	<0.001
A/REDF (%)	0	50	<0.001
Pathological Uterine Doppler (%)	0	66.67	<0.001
Labor (%)	63	17	*p* = 0.032
Antibiotics in labor (%)	31.58	83.33	*p* = 0.04
Delivery to processing (range in hours)	0–3	0–3	
Caesarean section (%)	37	83	*p* = 0.011
Anesthesia (%)	63.15	83.33	n.s.
Local	25	-	*p* = 0.044
Spinal	58.33	93.33	
Epidural	16.67	-	
General	-	6.67	
Maternal oxygen given at delivery? (%)	-	16.67	n.s.
Birth weight (g)	3550 (2920–4020)	AGA (n = 5): 2150 (1110–3180) FGR (n = 13): 1250 (610–1880)	<0.001
Placental weight (g)	580 (450–845)	310 (184–650)	<0.001
Fetal sex (%)			*p* = 0.061
Male	57.9	22.2	
Female	42.1	77.8	
Magnesium sulfate (%)	-	27.78	*p* = 0.02

## Supplementary Materials

Supplementary Materials are available online. Figure S1: HMGB1 expression levels in normal versus PE-AGA and PE-FGR placentae; Figure S2: HMGB1 expression levels in early-onset PE versus late-onset PE placentae; Figure S3: HMGB1 expression levels in control and PE placentas from women in labour and from women undergoing elective Caesarean sections (CS); Figure S4: 0,5 U LMWH effect on physiological term placental villous explants viability.
