# Supplementary material for: The HMGB1/RAGE Pro-Inflammatory Axis in the Human Placenta: Modulating Effect of Low Molecular Weight Heparin
Source: Molecules. 2017 Nov 17;22(11):1997. doi: 10.3390/molecules22111997 (PMC6150179; doi:10.3390/molecules22111997)
Supplement: Supplementary file 1 [file molecules-22-01997-s001.pptx]

## Slide 1
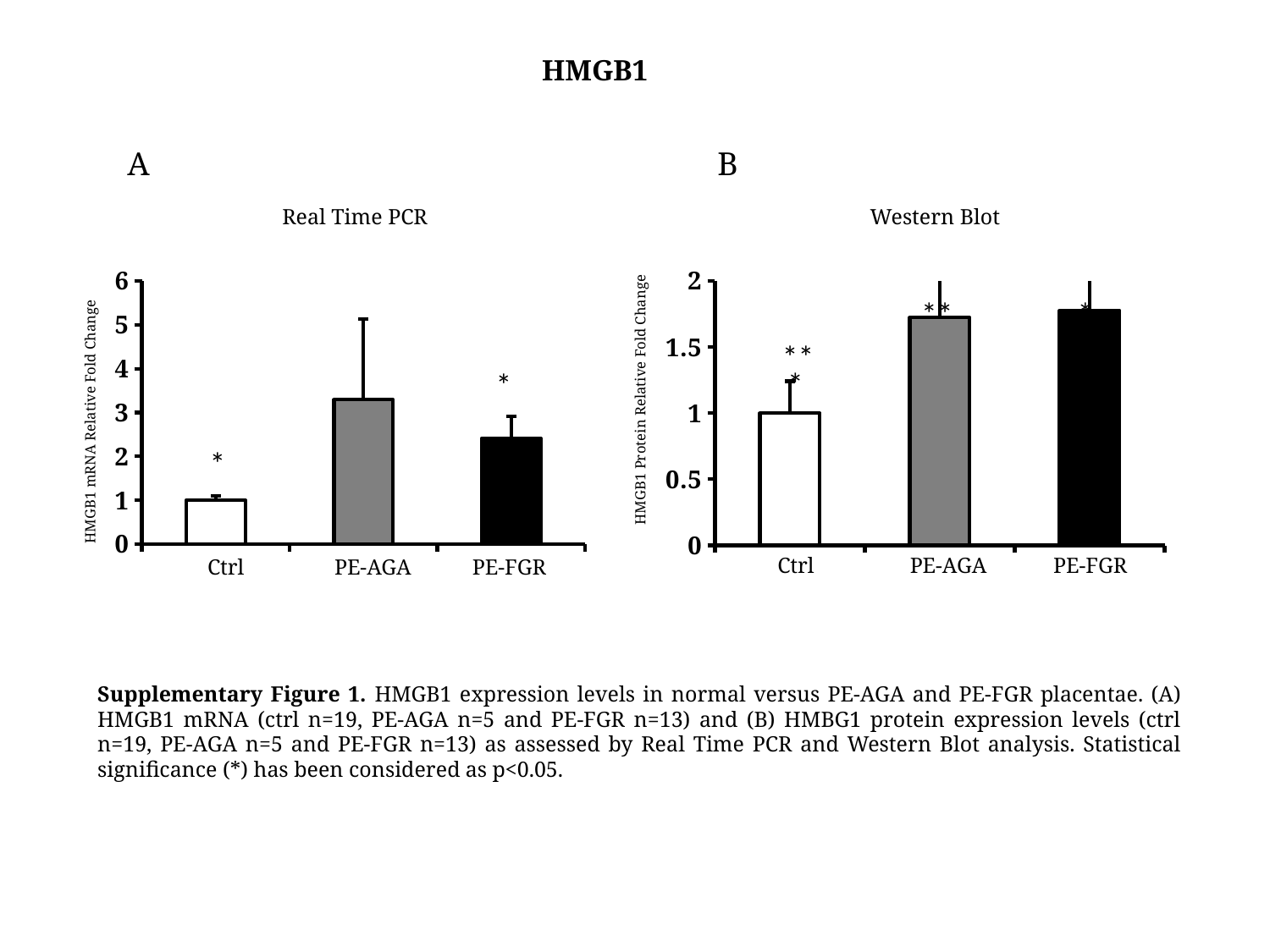

HMGB1
A
B
Real Time PCR
Western Blot
### Chart
| Category | |
|---|---|
| Fisio | 1.0 |
| PE- AGA | 3.2922979439599094 |
| PE-IUGR | 2.4100482267578385 |
### Chart
| Category | |
|---|---|
| ctrl | 1.0 |
| pe-aga | 1.7235675948590652 |
| pe-fgr | 1.7786697214665221 |**
*
**
*
*
HMGB1 Protein Relative Fold Change
HMGB1 mRNA Relative Fold Change
*
Ctrl	 PE-AGA	 PE-FGR
Ctrl	PE-AGA	 PE-FGR
Supplementary Figure 1. HMGB1 expression levels in normal versus PE-AGA and PE-FGR placentae. (A) HMGB1 mRNA (ctrl n=19, PE-AGA n=5 and PE-FGR n=13) and (B) HMBG1 protein expression levels (ctrl n=19, PE-AGA n=5 and PE-FGR n=13) as assessed by Real Time PCR and Western Blot analysis. Statistical significance (*) has been considered as p<0.05.

## Slide 2
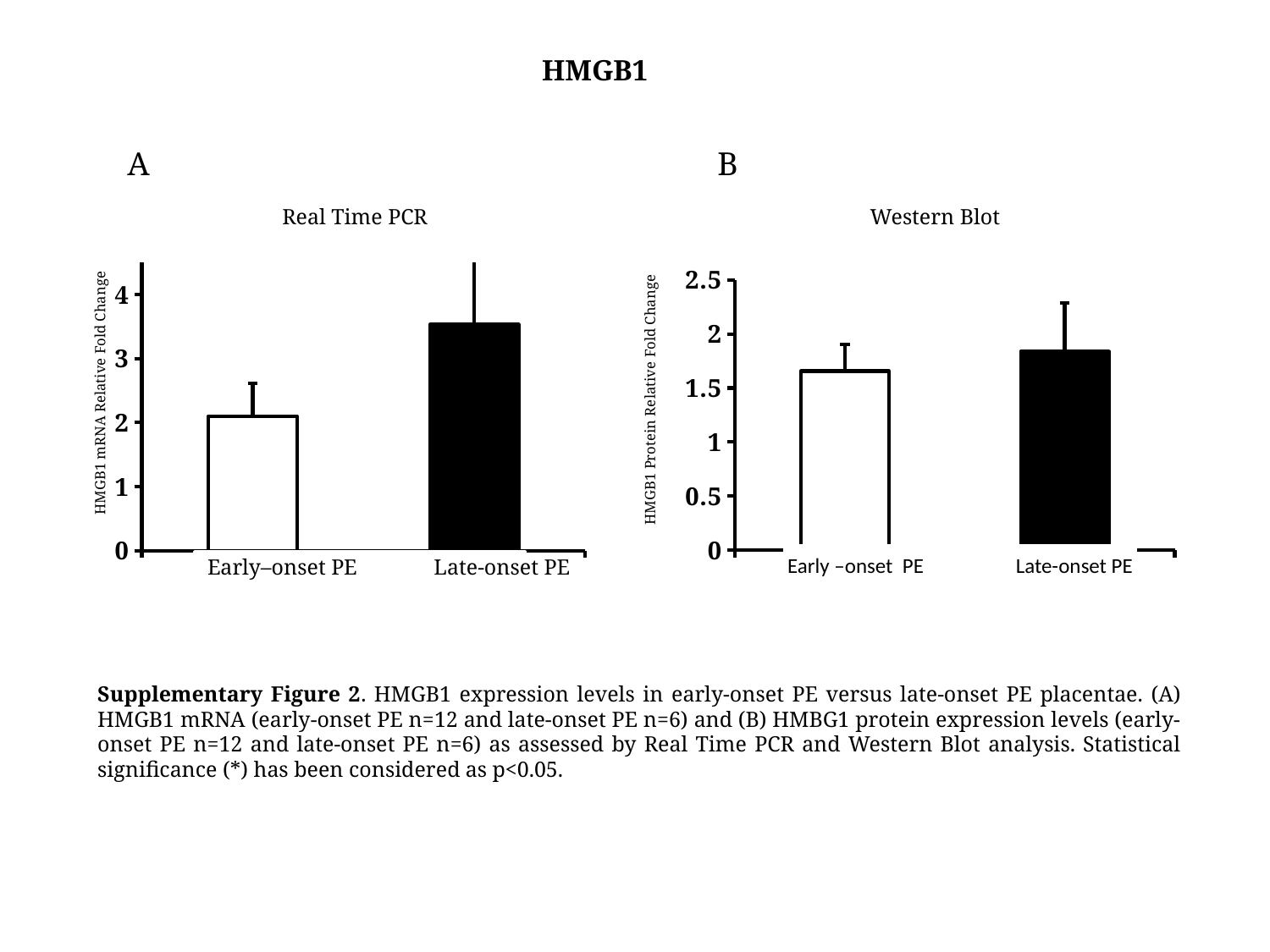

HMGB1
A
B
Real Time PCR
Western Blot
### Chart
| Category | MEDIA |
|---|---|
| PE before 34 | 2.102201967445246 |
| PE after 34 | 3.5335049036282813 |
### Chart
| Category | MEDIA |
|---|---|
| PE before 34 | 1.6575355217251706 |
| PE after 34 | 1.837469999999999 |HMGB1 mRNA Relative Fold Change
HMGB1 Protein Relative Fold Change
Early –onset PE Late-onset PE
Early–onset PE Late-onset PE
Supplementary Figure 2. HMGB1 expression levels in early-onset PE versus late-onset PE placentae. (A) HMGB1 mRNA (early-onset PE n=12 and late-onset PE n=6) and (B) HMBG1 protein expression levels (early-onset PE n=12 and late-onset PE n=6) as assessed by Real Time PCR and Western Blot analysis. Statistical significance (*) has been considered as p<0.05.

## Slide 3
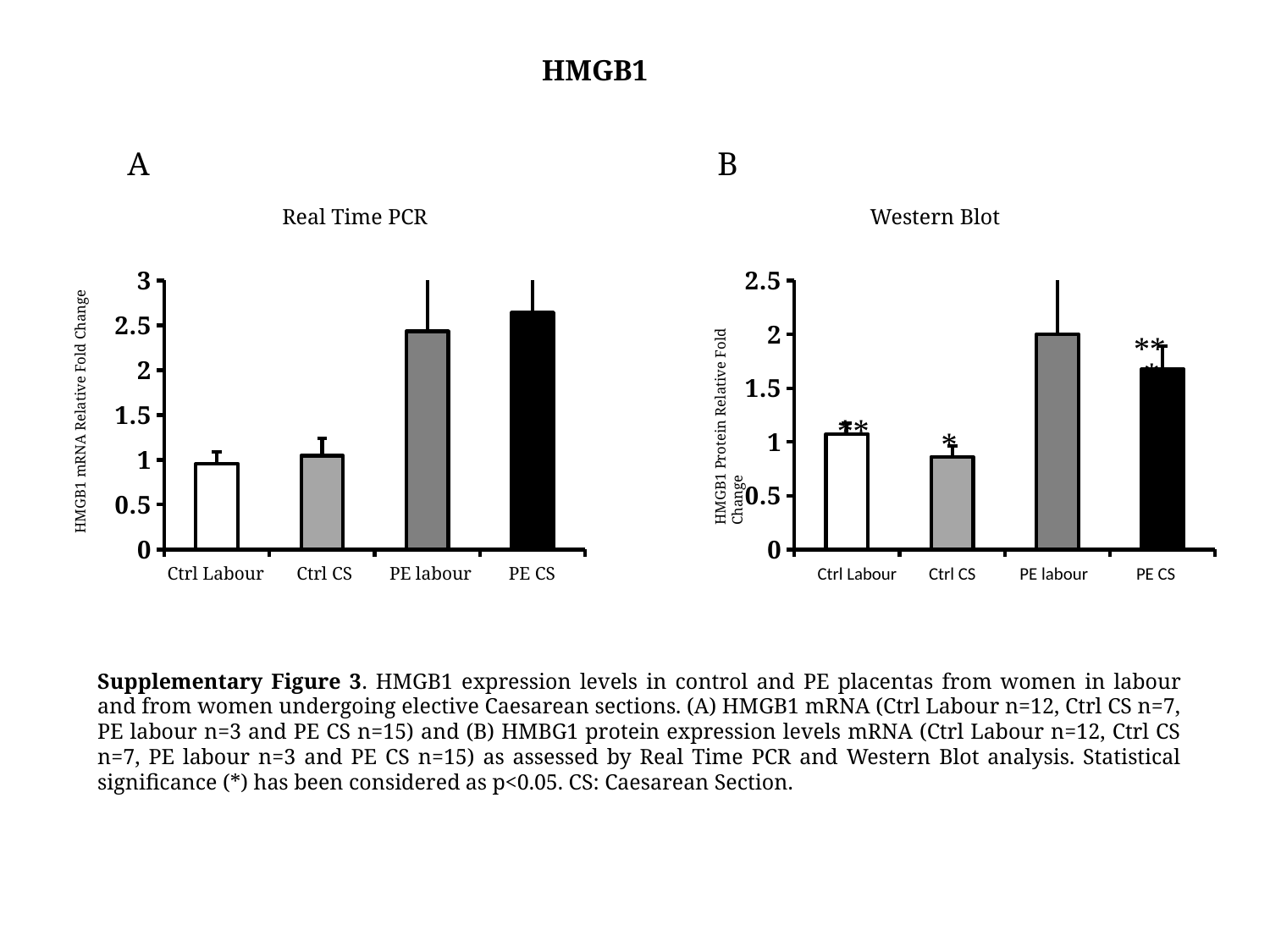

HMGB1
A
B
Real Time PCR
Western Blot
### Chart
| Category | |
|---|---|
| Fisio Labour | 0.9561173429145621 |
| Fisio Non Labour | 1.0469006376176364 |
| PE Labour | 2.4324735235575226 |
| PE Non Labour | 2.644030148323149 |
### Chart
| Category | |
|---|---|
| Fisio Labour | 1.0748398287312142 |
| Fisio Non Labour | 0.8627936473261101 |
| PE Labour | 2.0036777632440956 |
| PE Non Labour | 1.6753553950229874 |**
*
HMGB1 Protein Relative Fold Change
HMGB1 mRNA Relative Fold Change
**
*
Ctrl Labour Ctrl CS PE labour PE CS
Ctrl Labour Ctrl CS PE labour PE CS
Supplementary Figure 3. HMGB1 expression levels in control and PE placentas from women in labour and from women undergoing elective Caesarean sections. (A) HMGB1 mRNA (Ctrl Labour n=12, Ctrl CS n=7, PE labour n=3 and PE CS n=15) and (B) HMBG1 protein expression levels mRNA (Ctrl Labour n=12, Ctrl CS n=7, PE labour n=3 and PE CS n=15) as assessed by Real Time PCR and Western Blot analysis. Statistical significance (*) has been considered as p<0.05. CS: Caesarean Section.

## Slide 4
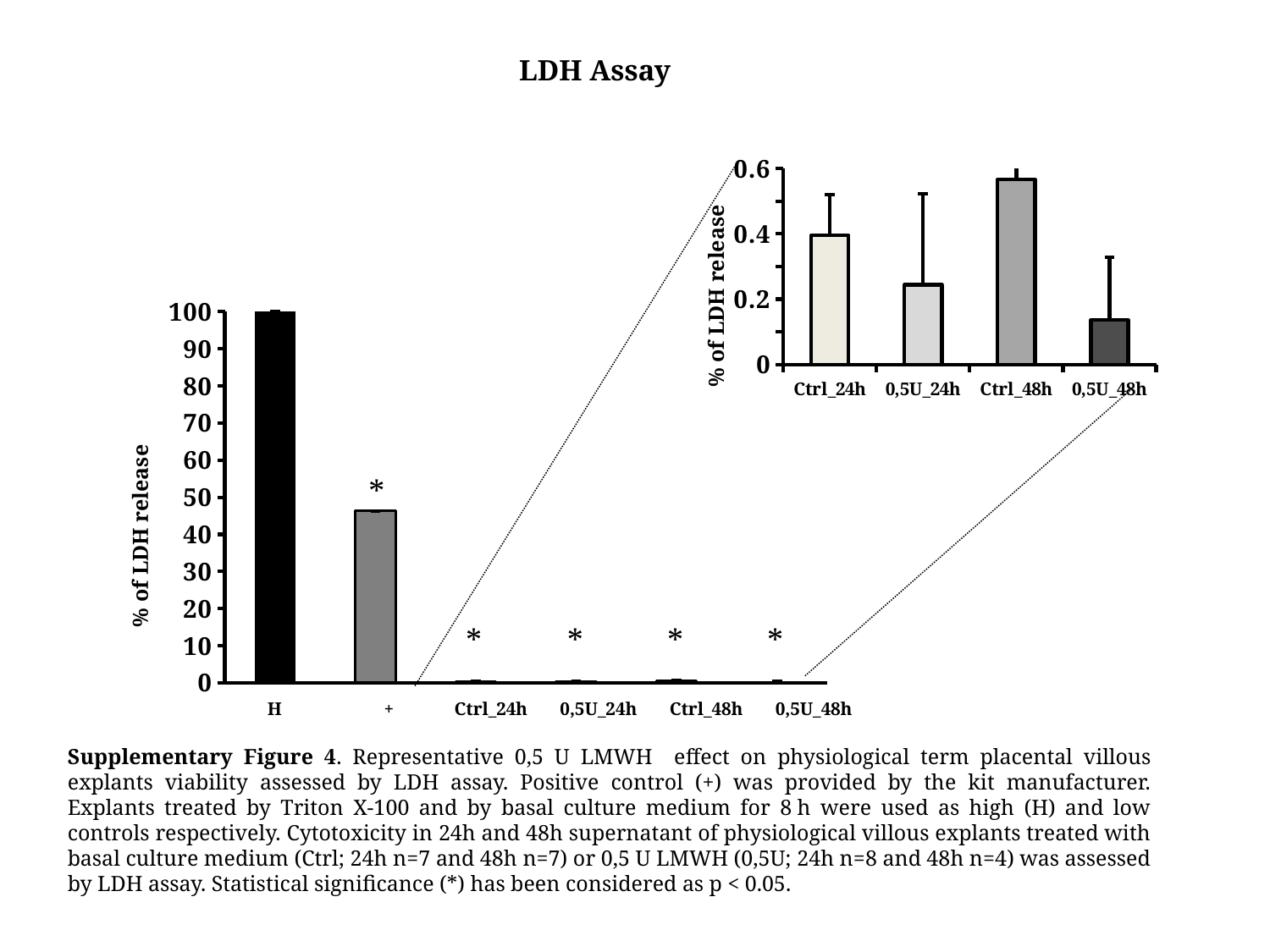

LDH Assay
### Chart
| Category | |
|---|---|
| Ctrl_24h | 0.3957441287141548 |
| 0,5U_24h | 0.24409627611262427 |
| Ctrl_48h | 0.5676657584014526 |
| 0,5U_48h | 0.13623978201634832 |% of LDH release
### Chart
| Category | |
|---|---|
| H | 100.0 |
| + | 46.321525885558586 |
| Ctrl_24h | 0.3957441287141548 |
| 0,5U_24h | 0.24409627611262427 |
| Ctrl_48h | 0.5676657584014526 |
| 0,5U_48h | 0.13623978201634832 |*
% of LDH release
*
*
*
*
H + Ctrl_24h 0,5U_24h Ctrl_48h 0,5U_48h
Supplementary Figure 4. Representative 0,5 U LMWH effect on physiological term placental villous explants viability assessed by LDH assay. Positive control (+) was provided by the kit manufacturer. Explants treated by Triton X-100 and by basal culture medium for 8 h were used as high (H) and low controls respectively. Cytotoxicity in 24h and 48h supernatant of physiological villous explants treated with basal culture medium (Ctrl; 24h n=7 and 48h n=7) or 0,5 U LMWH (0,5U; 24h n=8 and 48h n=4) was assessed by LDH assay. Statistical significance (*) has been considered as p < 0.05.
